# Specific Diagnosis

**Published:** 1892-02

**Authors:** 


Specific Diagnosis. A Study of Disease. With Special Reference to the Administration of Remedies. By John M. Scudder, M. D., Professor of Pathology and the Practice of Medicine in the Eclectic Medical Institute, Cincinnati, Ohio. Ninth Edition. John M. Scudder, Publisher, 1891. Price, $2.50.

